# Progranulin Facilitates Conversion and Function of Regulatory T Cells under Inflammatory Conditions

**DOI:** 10.1371/journal.pone.0112110

**Published:** 2014-11-13

**Authors:** Fanhua Wei, Yuying Zhang, Weiming Zhao, Xiuping Yu, Chuan-ju Liu

**Affiliations:** 1 Department of Orthopaedic Surgery, New York University Medical Center, New York, New York, United States of America; 2 Institute of Pathogenic Biology, Shandong University School of Medicine, Jinan, China; 3 Department of Cell Biology, New York University School of Medicine, New York, New York, United States of America; INSERMU1138, France

## Abstract

The progranulin (PGRN) is known to protect regulatory T cells (Tregs) from a negative regulation by TNF-α, and its levels are elevated in various kinds of autoimmune diseases. Whether PGRN directly regulates the conversion of CD4+CD25-T cells into Foxp3-expressing regulatory T cells (iTreg), and whether PGRN affects the immunosuppressive function of Tregs, however, remain unknown. In this study we provide evidences demonstrating that PGRN is able to stimulate the conversion of CD4+CD25-T cells into iTreg in a dose-dependent manner *in vitro*. In addition, PGRN showed synergistic effects with TGF-β1 on the induction of iTreg. PGRN was required for the immunosuppressive function of Tregs, since PGRN-deficient Tregs have a significant decreased ability to suppress the proliferation of effector T cells (Teff). In addition, PGRN deficiency caused a marked reduction in Tregs number in the course of inflammatory arthritis, although no significant difference was observed in the numbers of Tregs between wild type and PGRN deficient mice during development. Furthermore, PGRN deficiency led to significant upregulation of the Wnt receptor gene Fzd2. Collectively, this study reveals that PGRN directly regulates the numbers and function of Tregs under inflammatory conditions, and provides new insight into the immune regulatory mechanism of PGRN in the pathogenesis of inflammatory and immune-related diseases.

## Introduction

CD4+CD25+Foxp3+ regulatory T cells (Tregs) play a critical role in maintenance of peripheral tolerance and prevention of chronic inflammation and autoimmune diseases [1], [2]. Tregs can be divided into two main types: naturally occurring regulatory T cells (nTreg) and adaptive/inducible regulatory T cells (iTreg). nTreg are generated in thymus and represent a stable subpopulation and suppress the proliferation of self reactive T cells in the secondary lymphoid tissues [3]. In contrast, iTreg are generated in peripheral lymphoid tissues, which have variable expression of Foxp3 and may lose regulatory properties after their generation. Recent studies have shown that iTreg can be differentiated from the conventional CD4+CD25- T cells in the presence of TGF-β [4]. Since iTreg play essential roles in self-tolerance and autoimmunity, an investigation of iTreg induction and function would be of great importance in their therapeutic potential [5]–[7]. A global sequencing revealed that Foxp3 and Wnt target genes are considerably overlapped, suggesting a crucial role of Wnt signaling in Treg function [8]. In addition, stable expression of β-catenin enhanced the survival of Treg cells and rendered pathogenic CD4+CD25- T cells anergic [9].

Progranulin (PGRN), also called granulin epithelin precursor (GEP), PC-cell-derived growth factor (PCDGF), proepithelin, and acrogranin, is a 593-amino-acid secreted growth factor [10], [11]. PGRN is known to play an important role in a variety of physiologic and pathological processes, including wound healing, inflammation response, neurotrophic factor, and host defense [12]. PGRN can be induced in many cell types during inflammatory conditions, including immune cells and epithelial cells [13]. PGRN associates with some members in the TNF receptor superfamily, including TNFR1, TNFR2 and DR3 [12], [14]–[16], and possesses the ability to suppress inflammation in various kinds of conditions [12], [17]–[23]. The association between PGRN levels and systemic inflammation and autoimmunity has been reported [24]–[28], for instance, serum levels of PGRN were elevated in systemic lupus erythematosus and related with disease activity [25]. Auto-antibodies against PGRN have also been found in several autoimmune diseases, including rheumatoid arthritis, psoriatic arthritis, and inflammatory bowel disease, and such antibodies promoted a proinflammatory environment in a subgroup of patients [29]–[31]. Furthermore, PGRN was found to protect Tregs from a negative regulation by TNF-α [12], [30]. However, the direct regulation of PGRN on Tregs has not been reported yet. In this study, we present direct evidences that PGRN stimulates Tregs formation and is also required for its immunosuppressive activity.

## Materials and Methods

### Mice

All of the animal studies were performed and approved by the Institutional Animal Care and Use Committee of New York University (IACUC protocol #130202-01). C57BL/6, Foxp3-RFP mice (C57BL/6 background) were obtained from Jackson Laboratories. The generation of PGRN-deficient mice has been described previously [32]. All efforts were made to minimize animal suffering through anaesthesia.

### Flow cytometry

Spleen cells and lymphocytes from wild type and PGRN-deficient mice were stained using antibodies to mice CD4-FITC, CD25-PE and Foxp3-Alex Flour 647 (all from eBioscience, San Diego, CA, USA). Intracellular staining for Foxp3 (eBioscience, San Diego, CA, USA) were conducted according to the manufacturers' instructions. Data were acquired on a LSRII (BD) and analyzed with FlowJo (Tree star, Ashland OR). For CFSE labeling, cells were incubating with 5 mM CFSE (Invitrogen, Carlsbad, CA, USA) in PBS (containing 0.1% BSA) at 37°C for 10 min.

### Cell purification

For isolation of CD4+CD25- T cells, 1×10^7^ lymphocytes of spleens from 6- to 8-week-old mice were incubated with 10 µl antibody cocktail (Miltenyi Biotech, Bergisch Gladbach, Germany) and 10 µl anti-CD25 (biotin labeling), followed by incubation with 20 µl anti-biotin microbeads according to the manufactures instructions (Miltenyi Biotech, Bergisch Gladbach, Germany). The purity of the CD4+CD25- T cells was above 95%, as examined by FACS method.

For isolation of CD4+CD25+ T cells, lymphocytes of spleens from 6- to 8-week-old mice were depleted of cells that were labeled with Ab-to-mouse B220 and CD8 by magnetic cell sorting using Mitenyi reagents and a MACS apparatus. Then the lymphocytes were stained with CD4-APC and CD25-PE antibodies, washed 2 times with staining buffer and resuspended with sorting buffer. CD4+CD25+ T cells were sorted by FACS MoFlo cytometer. The purity of sorted fraction was 90–95%.

### PGRN immunization

The recombinant PGRN protein was prepared according to our previous publication [33]. One week-old Foxp3-RFP reporter mice were divided into two groups (n = 3). Two group mice were treated with 100 µg PGRN or PBS (serving as a control) by intraperitoneal injection every two days. After 1 week, the lymphocytes of spleen, peripheral lymph nodes (PLN), mesenteric lymph nodes (MLN), and Peyer's patches (PP) were collected for analysis by FACS.

### In vitro naïve CD4+CD25- T cells conversion assay

Naïve CD+CD25- T cells were purified from lymphocytes of spleens from Foxp3-GFP mice (6- to 8-week-old) using Mitenyi reagents and a MACS apparatus according to the manufactures instructions. Naïve cells were stimulated with plate-bound anti-CD3 (10 µg/ml, BD, San Diego, CA, USA) and soluble anti-CD28 (1 µg/ml, BD, San Diego, CA, USA) for 3–4 days in the presence of IL-2 (20 U/ml, R&D, Minneapolis, MN, USA). Human TGF-β (0 ng/ml, 0.01 ng/ml) and recombinant PGRN protein (0 µg/ml, 0.2 µg/ml, 1 µg/ml) were added as indicated. The induction of Foxp3+ T cells in the CD4+ fraction was detected by flow cytometry based on the levels of GFP.

### In vitro suppression assay

CD4+CD25- T cells were magnetic sorted from spleen lymphocytes of Thy1.1 mice (C57BL/6 background) using isolation kit from Miltenyi, and labeled with 5 mM CFSE as responder cells (Teff). 1×10^6^ spleen cells from TCRα-/-β-/- mice (C57BL/6 background) were lysed with red blood cell lysis buffer for 3 min, washed with pre-cooling PBS and treated with 1 µg mitomycin for 20 min, then resuspended with RPMI 1640 medium as antigen-presenting cells (APC). CD4+CD25+ T cells were FACS sorted from spleen lymphocytes of wild type and PGRN-deficient mice as suppressor cells.

For the analysis of suppression function, we performed assays in 96-well plate. Each well contained 0.5×10^5^ responder cells, 1×10^5^ mitomycin-treated APC cells and anti-CD3 at a concentration of 5 µg/ml. Suppressor cells were added at suppressor and responder cells rations of 1∶2, 1∶1, 2∶1, 4∶1 and 8∶1. Responder cells proliferation with wild type CD4+CD25+ T cells or PGRN-deficient CD4+CD25+ T cells were analyzed by FACS method assessing CFSE dilution after 3 days.

### BrdU incorporation assay

Wild type (n = 3) and PGRN-deficient mice (n = 3) were injected intraperitoneally with BrdU labeling reagent (BrdU, Sigma-Aldrich, St Louis, MO, USA) at a dose of 10 ml/kg body weight and sacrificed 2 hours later. Lymphocytes from spleen and lymph nodes were prepared. Then we performed cell surface staining with CD4-PerCP-cy5.5 and CD25-APC and permeabilized for intracellular staining of BrdU using the BrdU flow kit (BD, San Diego, CA, USA) according to the manufactures instructions.

### Real-time PCR

CD4+CD25+T cells were purified from the splenocytes of both wild type and PGRN-deficient mice by FACS sorting. Total RNA was extracted from CD4+CD25+T cells using RNeasy mini kit (Qiagen, Valencia, CA, USA). 1 µg RNA samples were reverse- transcribed by use of ImProm-II™ Reverse Transcription System (Promega, Madison, WI, USA). The primer pairs and expected length are in Table 1. Relative mRNA expression was measured as the fold increase in expression by 2-ΔΔct method and data was normalized to mRNA levels of GAPDH.

**Table 1:** Primer sequences.

Gene name	Primer sequence (5′-3′)	Product length (bp)
Wnt 1	Forward- ATTTTGCGCTGTGACCTCTT	
	Reverse- AGCAACCTCCTTTCCCACTT	177
Wnt 2	Forward- GGTCAGCTCTTCATGGTGGT	
	Reverse- GGAACTGGTGTTGGCACTCT	176
Wnt 3a	Forward- TCGGAGATGGTGGTAGAGAAA	
	Reverse- CGCAGAAGTTGGGTGAGG	130
Wnt 4	Forward- AAGAGGAGACGTGCGAGA AA	
	Reverse- CACCACCTTCCCAAAGACAG	191
Wnt 5a	Forward- CAAATAGGCAGCCGAGAGAC	
	Reverse- TGCAACCACAGGTAGACAGC	109
Wnt 5b	Forward- TCTCCGCCTCACAAAAGTCT	
	Reverse- CACAGACACTCTCAAGCCCA	242
Wnt 7a	Forward- GACAAATACAACGAGGCCGT	
	Reverse- GGCTGTCTTATTGCAGGCTC	207
Wnt 8b	Forward- CCAGAGTTCCGGGAGGTAG	
	Reverse- GAGATGGAGCGGAAGGTGT	131
Wnt 11	Forward- TGCTTGACCTGGAGAGAGGT	
	Reverse- AGCCCGTAGCTGAGGTTGT	193
Wisp 2	Forward- GTTTTGTGCCGCTGTGATG	
	Reverse- CTGAGGAGGGCTGGATTG	175
Fzd 1	Forward- TGCCCAGTGTCTTTCTCCTT	
	Reverse- TCTCTTTAGCCTCTCCCAACC	192
Fzd 2	Forward- ATCTGGAAACCTCCCAATCC	
	Reverse- CGTTTTGTTGCCCATTCTCT	184
Fzd 4	Forward- TGACAACTTTCACGCCGCTC	
	Reverse-ACAAGCCAGCATCGTAGCCACAC	397
Fzd 6	Forward- AGCCACCACACTCAGCTTTT	
	Reverse- CTACACTCTCCCTGCCCAAC	180
Fzd 7	Forward- AGAGACAAAGCGGGAAACAA	
	Reverse- TGTGCCTGAATGGGTATGAA	177
Fzd 8	Forward- TCCGTTCAGTCATCAAGCAG	
	Reverse- ATAGAAAAGGCAGGCGACAA	131
Fzd 9	Forward- GAAGCTGGAGAAGCTGATGG	
	Reverse- AAGTCCATGTTGAGGCGTTC	108
Fzd 10	Forward- GCTGCCCACATAACACACAC	
	Reverse- TCCTCACCCTCACTTGGTTC	179
β-catenin	Forward- TGAAGGCGTGGCAACATAC	
	Reverse- ATCAGGCAGCCCATCAACT	322
TCF 1	Forward- TCAAGAGGTGGGGGATTAGA	
	Reverse- GCAGGAGAAGCATTTGTAGG	185
TCF 3	Forward- ACCCCTTCCTGATGATTCC	
	Reverse- CGACCTTGTGTCCTTGACT	143
Wisp 1	Forward- CCCCTACAAGTCCAAGACCA	
	Reverse- TTTACCCTGAGCCACACACC	195
Axin 2	Forward- AACCTATGCCCGTTTCCTCT	
	Reverse- CCACACATTTCTCCCTCTCC	177
GAPDH	Forward-ACGTCGGTGGTAACGGATTTG	
	Reverse-TGTAGACCATGTAGTTGAGGTCA	123

### Induction of CIA

Chicken type II collagen (Chondrex, LLC, Seattle, WA) was emulsified with an equal volume of complete Freund's adjuvant (CFA) (Chondrex, LLC, Seattle, WA). Wild type mice (n = 6) and PGRN-deficient mice (n = 6) were intradermally immunized with 100 µl of the emulsion at the base of the tail. After 3 weeks, draining lymph nodes were extracted and CD4+CD25+Foxp3+ cells were analyzed by FACS.

### Statistical Analysis

Data was represented as mean ± SEM. Differences between the groups were analyzed with unpaired, 2-tailed t tests. P values less than 0.05 were considered as significant. All experiments were repeated two to three times with similar results.

## Results

### PGRN deficiency does not alter the numbers of CD4+CD25+Foxp3+ Treg cells in vivo

**Figure 1:**
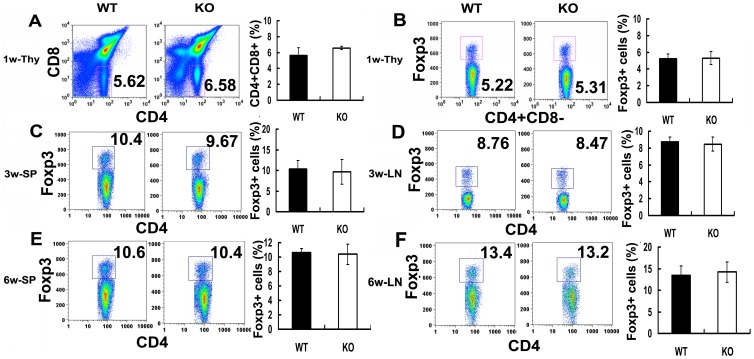
PGRN deficiency does not alter the generation of CD4+CD25+Foxp3+ T cells in vivo. Flow cytometric evaluation of CD4+CD25+Foxp3+ T cells in one-, three-, and six-week-old wild type (WT) and PGRN-deficient mice. (A) The percentage of CD4+ and CD8+ T cells in thymus from one-week-old C57BL/6 mice and PGRN-deficient mice. (B) The percentage of CD4+CD25+Foxp3+ cells in thymus from one-week-old C57BL/6 mice and PGRN-deficient mice. (C) The proportion of CD4+CD25+Foxp3+ cells in spleen from three-week-old C57BL/6 mice and PGRN-deficient mice. (D) CD4+CD25+Foxp3+ cells in lymph nodes from three-week-old C57BL/6 mice and PGRN-deficient mice. (E) The proportion of CD4+CD25+Foxp3+ cells in spleen from six-week-old C57BL/6 mice and PGRN-deficient mice. (F) CD4+CD25+Foxp3+ cells in lymph nodes from six-week-old C57BL/6 mice and PGRN-deficient mice. All data was representative of three mice per group and indicated as mean ± SEM.

To determine the role of PGRN in the development of naturally CD4+CD25+Foxp3+ cells, we used flow cytometry to analyze the numbers of CD4+CD25+Foxp3+ cells in lymphocytes of thymus, spleen, and lymph nodes from one-, three-, and six-week-old wild type and PGRN-deficient mice. As shown in Fig. 1A, one-week-old wild type and PGRN-deficient mice have comparable proportions of CD4+ and CD8+ T cells in thymus (*p*>0.05), and no significant difference in the percentage of CD4+CD25+Foxp3+ cells in thymus were found (*p*>0.05) (Fig. 1B). Equivalent numbers of CD4+CD25+Foxp3+ cells were found in the spleen and lymph nodes in three- and six-week-old PGRN-deficient mice, compared with wild type mice (*p*>0.05) (Fig. 1C–F). Thus, the results suggest that the absence of PGRN may not affect the generation of naturally CD4+CD25+Foxp3+ cells during development.

**Figure 2:**
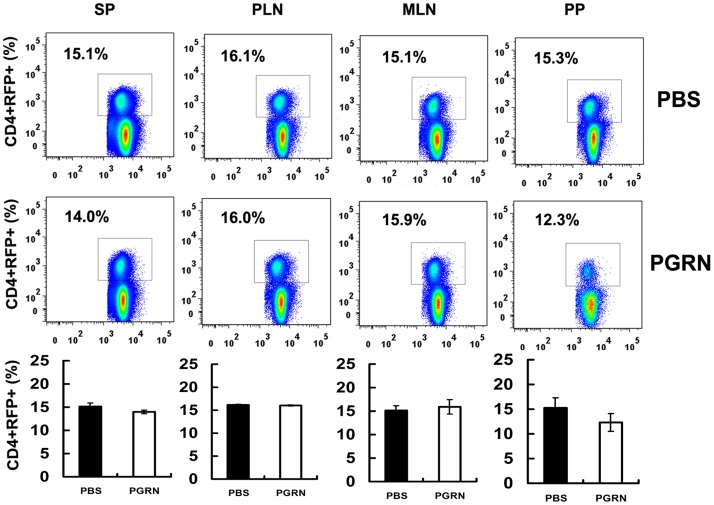
PGRN treatment does not change the proportions of CD4+CD25+Foxp3+ cells in normal conditions. One week-old Foxp3-RFP reporter mice were divided into two groups, three mice per group. PGRN group mice were treated with 100 µg PGRN every two days for 1 week, and PBS group mice were injected with the same volume of PBS as a control. The lymphocytes of spleen, peripheral lymph nodes (PLN), mesenteric lymph nodes (MLN), and Peyer's patches (PP) were isolated and analyzed by FACS. All data are representative of three independent experiments.

In a separate experiment, one-week-old Foxp3-RFP mice were treated with 100 µg PGRN every two days for 1 week, and the percentage of CD4+RFP+ cells in lymphoid tissues were analyzed by FACS. The results revealed that the numbers of CD4+RFP+ cells in spleen (15.1±0.8% RFP+ cells in PBS group versus 14.0±0.4% in PGRN group), peripheral lymph nodes (16.1±0.2% RFP+ cells in PBS group versus 16.0±0.1% in PGRN group), mesenteric lymph nodes (15.1±1.0%RFP+ cells in PBS group versus 15.9±1.6% in PGRN group), and Peyer's patches (15.3%±2.0 RFP+ cells in PBS group versus 12.3±1.8% in PGRN group) in these two groups was not significantly changed (*p*>0.05) (Fig. 2). In brief, these findings demonstrate that PGRN treatment does not change the proportions and numbers of CD4+CD25+Foxp3+ cells under physiological conditions.

### PGRN promotes the CD4+CD25- T cells conversion into Foxp3-expressing iTreg

**Figure 3:**
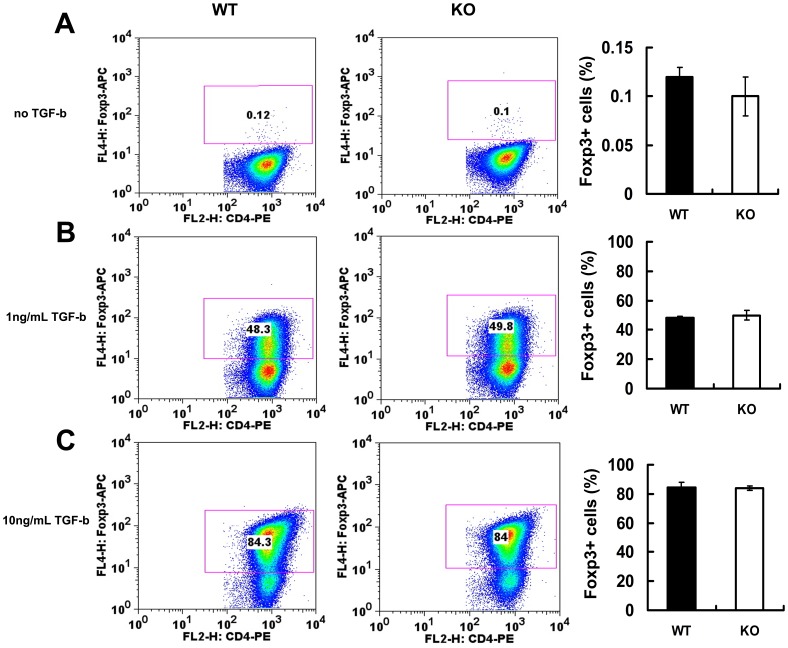
PGRN deficiency does not affect the conversion of naïve CD4+CD25- T cells into iTreg mediated by TGF-β. Naïve CD4+ CD25- T cells isolated from both wildtype (WT) and PGRN-deficient mice were stimulated with plate-bound CD3 Ab and solute CD28 Ab and cultured the cells for 3-4 days in the presence or absence of TGF-β, and the expression of GFP was measured by FACS. (A) The GFP levels in CD4+ T cells in the absence of TGF-β. (B) The GFP levels in CD4+ T cells in the presence of 1 ng/ml TGF-β. (C) The GFP levels in CD4+ T cells in the presence of 10 ng/ml TGF-β. All data were repeated three times with similar results.

Since iTreg cells are essential in immune tolerance and in the prevention of chronic inflammation [34], growth factors which can boost TGF-β-mediated the conversion of CD4+CD25- T cells into iTreg will be of great importance in inflammatory conditions. TGF-β was reported to induce Foxp3-expressing iTreg [35]. We sought to determine whether PGRN regulates the conversion of CD4+CD25- T cells into iTreg. In a loss-of-function study, we first stimulated naïve wild type and PGRN-deficient CD4+CD25- T cells with plate-bound CD3 antibody and soluble CD28 antibody in the presence of IL-2 and cultured for 3-4 days with or without TGF-β. In the absence of TGF-β, PGRN-deficient CD4+CD25- T cells have comparable capacity to convert into iTreg cells (0.10±0.02% Foxp3+ cells in KO versus 0.12±0.01% in WT, *p*>0.05) (Fig. 3A). In addition, no difference were found in wild type and PGRN-deficient CD4+CD25- T cells conversion into iTreg cells in the presence of TGF-β at dose of 1 ng/ml (49.8±3.3% Foxp3+ cells in KO versus 48.3±1.0% in WT, *p*>0.05) and 10 ng/ml (84±1.4% Foxp3+ cells in KO versus 84.3±3.5% in WT, *p*>0.05) (Fig. 3B-C). Thus, the findings suggest endogenous PGRN may not be required for CD4+CD25- T cells conversion into iTreg.

**Figure 4:**
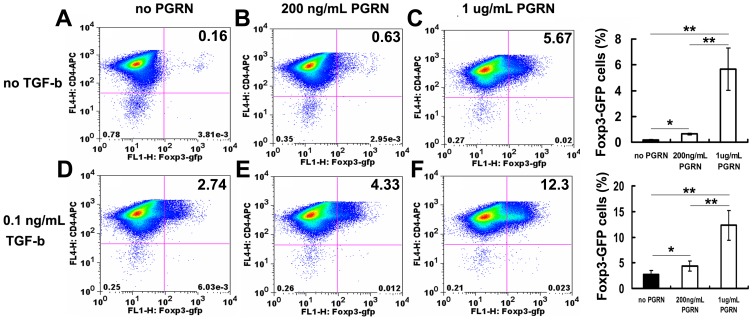
Recombinant PGRN promotes the induction of inducible regulatory T cells in vitro. Naïve CD4+GFP- cells in lymphocytes of spleen from Foxp3-GFP reporter mice were purified by using Mitenyi reagents and a MACS apparatus. The purity of cells was evaluated by FACS method. CD4+GFP- cells conversion assay was performed as described in Methods, and GFP expression in TGF-β-unstimulated CD4+GFP- cells was taken as a control. After 3-4 days, cells were washed and GFP expression was analyzed by FACS. Data represent three independent experiments are shown. (A) No TGF-β and no PGRN. (B) No TGF-β plus 200 ng/ml of PGRN. (C) No TGF-β plus 1 µg/ml of PGRN. (D) 0.1 ng/ml of TGF-β and no PGRN. (E) 0.1 ng/ml of TGF-βplus 200 ng/ml of PGRN. (F) 0.1 ng/ml of TGF-β plus 1 µg/ml of PGRN. Data represent as a means ±SE of a representative experiment. *p<0.05; **p<0.01.

We further performed the gain-of-function experiment, we treated CD4+GFP- T cells with different concentrations of PGRN and examined the change of GFP expression in CD4+ T cells. As shown in Fig. 4, PGRN significantly promoted the conversion of CD4+GFP- T cells into CD4+GFP+ T cells at a dose-dependent manner in the presence of 0.1 ng/ml TGF-β (2.74±0.70% GFP+ cells with no PGRN versus 4.33±0.98% GFP+ cells with 200 ng/ml PGRN versus 12.3±2.85% GFP+ cells with 1 µg/ml PGRN, *p* <0.05) (Fig. 4D–F). Furthermore, in the absence of TGF-β, 1 µg/ml of PGRN significantly induced the expression of GFP in 5.67±1.65% of the cells, when compared with 0.16±0.07% GFP+ cells without PGRN (*p* <0.01) (Fig. 4A and C). And low concentrations of PGRN also significantly induced GFP expression in CD4+ cells, when compared with no PGRN conditions (0.63±0.23% GFP+ cells versus 0.16±0.07% GFP+ cells, *p* <0.05) (Fig. 4B). Taken together, these results indicate that recombinant PGRN promotes and synergistically enhances TGF-β-mediated induction of inducible regulatory T cells *in vitro*.

### PGRN deficiency decreases the immunosuppressive function of CD4+CD25+ T cells

**Figure 5:**
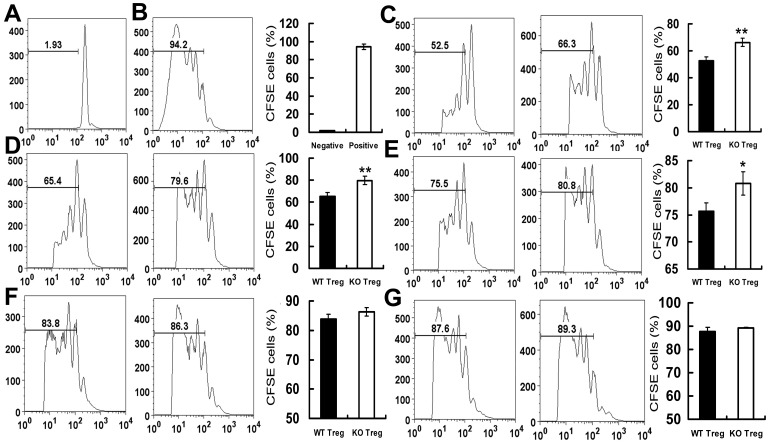
PGRN-deficient CD4+CD25+ Treg decreased the suppressive capacity to Teff proliferation. Freshly isolated, CFSE-labeled CD4+CD25- T cells from Thy1.1 mice were used as Teff and co-cultured with Tregs at ratios of 1∶2, 1∶1, 2∶1, 4∶1 and 8∶1. CFSE-based Teff proliferation suppression assay in vitro in which 5×10^5^ CFSE-labeled Teff cells were stimulated with CD3 antibody in the presence of mitomycin-treated APC cells and different ratios of FACS sorted wild type or PGRN-deficient Tregs. The CFSE proliferation was evaluated by FACS. All data are representative of three separate experiments. (A) Negative control. (B) Positive control. (C) The CFSE dilution when Teff co-cultured with Tregs at ratios of 1∶2. (D) The CFSE dilution when Teff co-cultured with Tregs at ratios of 1∶1. (E) The CFSE dilution when Teff co-cultured with Tregs at ratios of 2∶1. (F) The CFSE dilution when Teff co-cultured with Tregs at ratios of 4∶1. (G) The CFSE dilution when Teff co-cultured with Tregs at ratios of 8∶1. Data represent as a means ±SE of a representative experiment. *p<0.05; **p<0.01.

The proportion of normal CD4+CD25+ Tregs constitutes 5–10% of peripheral CD4+ T cells in mice and 1–2% in humans, and can potently suppress the proliferation of active CD4+CD25- and CD8+ T cells [36], [37]. To evaluate the role of PGRN signaling in regulation of CD4+CD25+ Tregs function, we performed in vitro CFSE-based proliferation suppression assay. 5×10^5^ CFSE-labeling Teff cells were stimulated for 72 hours with CD3 antibody (5 µg/ml) in the presence of 1×10^5^ APC cells and varying ratios of FACS purified WT or PGRN-deficient CD4+CD25+ Tregs, and CFSE dilution was evaluated by FACS. The CFSE proliferation in negative control and positive control group was 1.93±0.1% and 94.2±3.2%, respectively (Fig. 5A and B). Our results demonstrate that wild type and PGRN-deficient CD4+CD25+ Tregs significantly suppress the CFSE proliferation when Teff co-cultured with Tregs at rations of 1∶2, 1∶1, 2∶1 and 4∶1, compared with positive control group (*p*<0.05) (Fig. 5B–F). PGRN-deficient Tregs significantly decreased suppressive capacity when Teff co-cultured with Tregs at ratios of 1∶2 (66.3±3.2% CFSE dilution in KO versus 52.5±3.0% CFSE dilution in WT, *p*<0.01, Fig. 5C) and 1∶1 (79.6±3.78% CFSE dilution in KO versus 65.4±3.6% CFSE dilution in WT, *p*<0.01, Fig. 5D), compared with wild type Tregs. Suppressor cells were added at suppressor and responder cells rations of 2∶1, PGRN-deficient Tregs showed a slightly lower suppressive capacity than wild type Tregs (80.8±2.18% CFSE dilution in KO versus 75.5±1.5% CFSE dilution in WT, *p*<0.05, Fig. 5E). However, no significant difference were found between wild type and PGRN-deficient Tregs at ratios of 4∶1 (86.3±1.4% CFSE proliferation in KO versus 83.8±1.7% CFSE proliferation in WT, *p*>0.05, Fig. 5F) and 8∶1 (89.3±0.1% CFSE dilution in KO versus 87.6±2.0% CFSE dilution in WT, *p*>0.05, Fig. 5G). Collectively, PGRN is required for the immunosuppressive function of Tregs *in vitro*.

### PGRN deficiency does not affect the proliferation of CD4+CD25+ Treg in vivo

**Figure 6:**
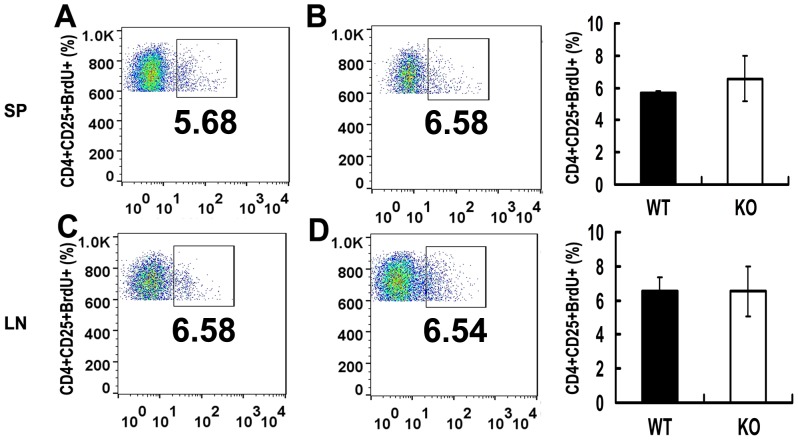
In vivo proliferation of CD4+CD25+ T cells from spleen and lymph nodes analyzed by BrdU corporation. Wild type and PGRN-deficient mice were injected intraperitoneally with BrdU labeling reagents, and the degrees of BrdU incorporation by CD4+CD25+ T cells from lymphocytes of spleen and lymph nodes were determined by FACS. (A) The BrdU incorporation of wild type CD4+CD25+ T cells from splenocytes. (B) The BrdU incorporation of PGRN-deficient CD4+CD25+ T cells from splenocytes. (C) The percentage of wild type CD4+CD25+ T cells from lymphocytes of lymph nodes that incorporated BrdU. (D) The percentage of PGRN-deficient CD4+CD25+ T cells from lymphocytes of lymph nodes that incorporated BrdU. All data was representative of three mice per group and indicated as mean ±SEM.

5-Bromo-2-deoxyuridine (BrdU) is a pyrimidine analogue of thymidine, selectively incorporated into replicating DNA, effectively tagging dividing cells. To determine whether PGRN deficiency alters the proliferation of Tregs *in vivo*, we set a BrdU incorporation assay. We injected BrdU labeling reagent (BrdU, Sigma-Aldrich) at a dose of 10 ml/kg body weight into wild type and PGRN-deficient mice, three mice per group. Mice were sacrificed 2 hours after injection and intracellular staining of BrdU in lymphocytes of spleen (SP) and lymph nodes (LN) were stained with BrdU flow kit (BD Bioscience) and analyzed by FACS. We did not observe any significant changes in the number of CD4+CD25+BrdU+ cells from splenocytes between wild-type and PGRN-deficient mice (6.58±1.42% BrdU+ cells in KO versus 5.68±0.11% BrdU+ cells in WT, *p*>0.05) (Fig. 6A–B). In addition, 6.54±1.46% of the cells in lymphocytes of lymph nodes are BrdU positive from PGRN-deficient mice, and comparable to 6.58±0.77% BrdU+ cells seen in wild type mice (*p*>0.05) (Fig. 6C and D). These findings suggest that wild type and PGRN-deficient CD4+CD25+ Treg cells have a comparable proliferation and division capacity *in vivo*.

### PGRN deficiency leads to fewer Treg cells in collagen-induced arthritis (CIA)

**Figure 7:**
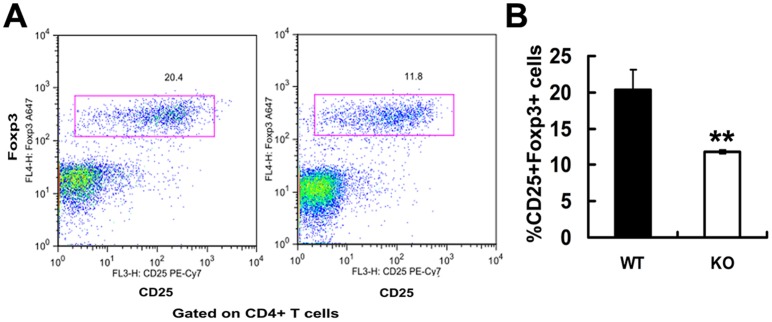
Fewer CD4+CD25+Foxp3+ Treg cells seen in PGRN-deficient CIA model. Wild type (n = 6) and PGRN-deficient mice (n = 6) were intradermally immunized with 100 µl of chicken type II collagen emulsified with an equal volume of complete Freund's adjuvant (CFA). 21 days post immunization, draining lymph nodes were extracted and CD4+CD25+Foxp3+ T cells were analyzed by FACS. Data represent as a means ±SE of a representative experiment. **p<0.01.

To determine whether the PGRN deficiency alters the number of Tregs in inflammatory conditions, we established a collagen-induced arthritis (CIA) model. Wild type and PGRN-deficient mice, six mice per group, were intradermally injected with 100 µl of the emulsion at the base of the tail. Two group mice were sacrificed 21 days after immunization and intracellular staining of Foxp3 in lymphocytes of draining lymph nodes (LN) was stained and analyzed by FACS. The results demonstrate that PGRN-deficient CD4+CD25- T cells have an impaired ability to generate iTreg in arthritis conditions (Fig. 7A–B). Arthritic PGRN-deficient mice shown a significant changes in the number of CD4+CD25+Foxp3+ cells from draining lymph nodes (11.8±0.2% CD4+CD25+Foxp3+ cells in arthritic KO mice versus 20.4±2.7% CD4+CD25+Foxp3+ cells in arthritic WT mice, *p*<0.01, Fig. 7A–B). These findings suggest that PGRN deficiency leads to fewer CD4+CD25+Foxp3+ Treg cells in collagen-induced arthritis conditions.

### PGRN deficiency upregulates the expression of Fzd2 in CD4+CD25+ T cells

**Figure 8:**
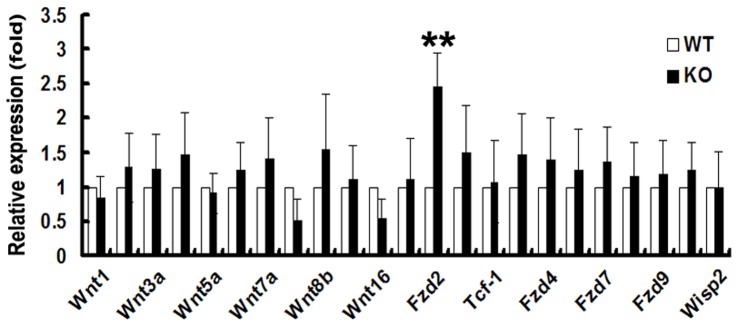
Wnt signaling components expression in wild type and PGRN-deficient CD4+CD25+ T cells measured by real-time PCR. All values are shown as a relative ratio to GAPDH measured by 2-ΔΔct method. Data represent as a means ±SE of a representative experiment. *p<0.05; **p<0.01.

Wnt signaling proteins can be divided into two subgroups according to the downstream molecules, the canonical pathway which stabilized β-catenin and activated target genes through the regulation of TCF/Lef transcription factors, and the noncanonical pathway did not dependent on the regulation of β-catenin and activated protein kinase C and G proteins, etc [38]. It was reported that Wnt signaling regulated PGRN-mediated frontotemporal dementia (FTD) and PGRN and Wnt reciprocally regulated each other [38], [39]. Moreover, Wnt signaling was also reported to stabilize the survival of CD4+CD25+ Treg cells and to enhance their suppressive capacity [9], [40]. To further study the molecular events underlying PGRN-mediated regulation of CD4+CD25+ T cells, we purified CD4+CD25+ T cells from splenocytes of wild type and PGRN-deficient mice by FACS sorting and examined the gene expression of Wnt signaling components through real-time PCR. Our results did not found significantly change of Wnt1, Wnt2, Wnt3a, Wnt4, Wnt5a, Wnt5b, Wnt7a, Wnt8b, Wnt11, Wisp2, Fzd1, Fzd4, Fzd6, Fzd7, Fzd8, Fzd9, Fzd10, β-catenin, TCF1, TCF3, wisp1 and axin2 gene expression (*p*>0.05) (Fig. 8). Interestingly, the mRNA level of Fzd2 gene in PGRN-deficient CD4+CD25+ T cells was significantly upregulated, when compared with its expression in wild type CD4+CD25+ T cells (*p*<0.01) (Fig. 8). Collectively, this set of experiments indicated that PGRN deficiency upregulates the expression of Fzd2 gene in CD4+CD25+ T cells.

## Discussion

Inducible CD4+CD25+Foxp3+ regulatory T cells (iTreg) develop outside of the thymus and play an essential role in controlling of chronic inflammation and autoimmunity [34]. Therefore, investigation of the growth factors which can convert naïve conventional T cells into iTreg may provide a new strategy for manipulating chronic inflammation and autoimmune diseases. In this study, we examine the role of PGRN in the conversion of CD4+CD25- T cells into Foxp3-expressing iTreg and immunosuppressive function of CD4+CD25+ Tregs. Our findings demonstrate that PGRN significantly promotes the conversion of naïve CD4+CD25- T cells into iTreg mediated by lower concentration of TGF-β in a dose-dependent manner (Fig. 4D–F). PGRN alone also effectively induce the generation of iTreg, although less efficiently than the conversion capacity induced by TGF-β (Fig. 4A–C). The findings that PGRN alone or combined with TGF-β stimulates the production of iTreg may provide new insights into the conversion of naïve CD4+CD25- T cells into iTreg.

PGRN deficiency does not alter the numbers and percentage of CD4+CD25+Foxp3+ T cells in thymus, spleen, and lymph nodes in different ages of mice (Fig. 1). In addition, mice in PGRN-treated group have a comparable number of CD4+CD25+Fxop3+ T cells in spleen, peripheral lymph nodes, mesenteric lymph nodes, and Peyer's patches, when compared with the PBS group (Fig. 2). However, PGRN deficiency leads to a marked reduction of Treg number in collagen-induced inflammatory arthritis (Fig. 7). These results suggest that PGRN is important for the Tregs formation under inflammatory conditions, and does not influence the development of Tregs under normal immune homeostasis. A deficiency or defective function of Tregs is common in autoimmune diseases such as rheumatoid arthritis [41]. Furthermore, therapeutic agents that target Tregs can benefit rheumatoid arthritis. For instance, intravenous immunoglobulin (IVIg) also induces the expansion of Tregs and enhances their suppressive function and exerts beneficial effect in autoimmune diseases [42]–[46]. In addition, our previous report also supports this concept [12]. In CIA model, PGRN inhibits Th1 (IFNγ) cytokines production in Teff cells, decreases the levels of IL-6 expression in serum, prevents TNFα-induced downregulation of Tregs suppressive function and inhibits inflammatory arthritis in mice [12].

CD4+CD25+ Tregs potently suppress the proliferation of active CD4+CD25- cells [36], [37]. In vitro Teff proliferation suppression assay demonstrated that PGRN deficiency led to significant reduction in the suppressive function of Tregs (Fig. 5C–E), indicating an important immunosuppressive role of PGRN in Tregs. PGRN insufficiency resulted from the mutations in the *GRN* gene was reported to cause reduced survival signaling and accelerated cell death in neurons [47]–[49]. PGRN deficiency does not affect the proliferation of Teff cells (data not show). Therefore, we further investigated the correlation between Tregs function and cell survival in PGRN-deficient mice using BrdU incorporation assay. Interestingly, we did not observe significant difference in CD4+CD25+BrdU+ numbers between wild type and PGRN-deficient mice (Fig. 6A–D), suggesting PGRN-deficiency may not impair Tregs survival and proliferation under normal immune homeostasis *in vivo*.

It is known that Wnt signaling plays an important role in regulating CD4+CD25+ Tregs. For instance, β-catenin and Wnt3a both regulate Tregs function [8], [9], [40]. Fzd2 receptor was reported to be involved in the Wnt3a-dependent activation of β-catenin pathway and also required for Wnt5a-mediated β-catenin-independent pathway [50]. In our study, we found the level of Fzd2 was upregulated in PGRN-deficient Treg cells (Fig. 8). The finding is consistent with a recent report that Fzd2 is upregulated in PGRN-knockout mice using weighted gene coexpression network analysis (WGCNA) [39]. It is postulated that regulation of Fzd2 by PGRN may also contribute to the PGRN-mediated regulation of Tregs.

PGRN associates with some members in the TNF receptor superfamily, including TNFR1, TNFR2 and DR3 [12], [14]–[16], and possesses the ability to suppress inflammation in various kinds of conditions [12], [17]–[23]. Auto-antibodies against PGRN have been found in several autoimmune diseases, including rheumatoid arthritis, psoriatic arthritis, and inflammatory bowel disease, and such antibodies promoted a proinflammatory environment in a subgroup of patients [29]–[31]. In accordance with the finding that PGRN binds to TNFR, we found that PGRN protected Tregs from a negative regulation by TNF-α [12]. This finding has been also independently confirmed by other laboratories [30]. Chen and colleagues agreed that PGRN played an protective role in Tregs, but through enhancing TNF-α-induced Tregs proliferation [51]. The effect of TNF-α on the regulation of Tregs purified from mice and humans appears to be highly controversial. The data from Chen lab suggest that TNF-α promotes murine Tregs activity *in vitro* [51], whereas in humans, TNF-α inhibits the suppressive function of Tregs through negative regulation of Foxp3 expression [30], [52]–[55]. Although the effect of TNF-α on Tregs function remains controversy, the beneficial and therapeutic effects of Tregs in autoimmune diseases have been well-accepted by the scientific community [56], [57]. In addition, TNF-α inhibitors have been accepted as the most effective anti-inflammatory therapeutics.

In summary, this study provides evidences demonstrating that PGRN directly regulates the induction of iTreg and function of Tregs *in vitro*, in addition to its antagonizing TNF-α-mediated negative regulation of Tregs. More importantly, PGRN deficiency leads to a significant reduction in Tregs in the course of inflammatory arthritis *in vivo*. Additionally, selective and significant upregulation of Fzd2 gene expression in PGRN deficient Tregs may contribute to the PGRN regulation of Tregs. These findings not only provide new insights into the role and regulation of PGRN in Tregs, but also present PGRN and/or its derivatives as therapeutic targets for treating chronic inflammatory and autoimmune diseases.
